# *ELMOD3*‐*SH2D6* gene fusion as a possible co‐star actor in autism spectrum disorder scenario

**DOI:** 10.1111/jcmm.14733

**Published:** 2019-12-04

**Authors:** Eleonora Loi, Loredana Moi, Sylvain Blois, Elena Bacchelli, Ana Florencia Vega Benedetti, Cinzia Cameli, Roberta Fadda, Elena Maestrini, Marinella Carta, Giuseppe Doneddu, Patrizia Zavattari

**Affiliations:** ^1^ Department of Biomedical Sciences Unit of Biology and Genetics University of Cagliari Cagliari Italy; ^2^ Department of Pharmacy and Biotechnology University of Bologna Bologna Italy; ^3^ Department of Pedagogy, Psychology, Philosophy University of Cagliari Cagliari Italy; ^4^ Center for Pervasive Developmental Disorders AO Brotzu Cagliari Italy

**Keywords:** autism spectrum disorder, copy number variant, *ELMOD3*, gene fusion, *SH2D6*

## Abstract

Autism spectrum disorder (ASD) is a group of neurodevelopmental disorders characterized by high heritability. It is known that genetic factors contribute to ASD pathogenesis. In particular, copy number variants (CNVs) are involved in ASD susceptibility and can affect gene expression regulation. 2p11.2 microdeletions encompassing *ELMOD3*, *CAPG* and *SH2D6* genes have been described in four unrelated ASD families. The present study revealed that this microdeletion is responsible for the production of a chimeric transcript generated from the fusion between *ELMOD3* and *SH2D6*. The identified transcript showed significantly higher expression levels in subjects carrying the deletion compared to control subjects, suggesting that it is not subjected to nonsense‐mediated decay and might encode for a chimeric protein. In conclusion, this study suggests the possible involvement of this gene fusion, together with the other previously identified variants, in ASD.

## 1 INTRODUCTION

Copy number variants (CNVs) are DNA segments larger than 1 kilobase and present at variable copy number compared to a reference genome. The involvement of CNVs on Autism spectrum disorder (ASD) susceptibility is well‐established.^1^, ^2^, ^3^ In fact, ASD individuals show an increased burden of genic CNVs compared to control subjects.^4^ CNVs can affect gene expression, therefore, contributing to disease pathogenesis, by several mechanisms, including gene dosage, gene interruption, position effects, gene fusion and unmasking of recessive alleles or polymorphisms.^5^

Gene fusions are well studied in cancer genetics, as chimeric genes have an important role in different cancers. They can occur when CNV breakpoints disrupt two genes encoded on the same chromosomal strand.

Some studies have previously investigated whether CNVs generating fusion transcripts may have an impact on ASD susceptibility^6^ obtaining negative results. However, fusion transcripts were not detected for 80% of CNVs tested and the only CNV resulting in a fusion transcript was present in ASD and control subjects at similar rate.^6^ In another study, Pagnamenta et al detected a *DOCK4*‐*IMMP2L* fusion transcript in both ASD subjects and their non‐affected family members. This fusion transcript was expressed at very low levels compared to the wild‐type transcript. In fact, *IMMP2L* portion of the fusion gene was out of frame leading to a premature stop codon and likely to nonsense‐mediated decay of the fusion transcript.^7^

Another indirect way, by which CNVs can alter gene expression, is the disruption of a gene regulatory landscape. Gene transcription is regulated by cis‐acting elements that can be separated from their target gene by the coding sequences of neighbouring genes.^8^

**Figure 1:**
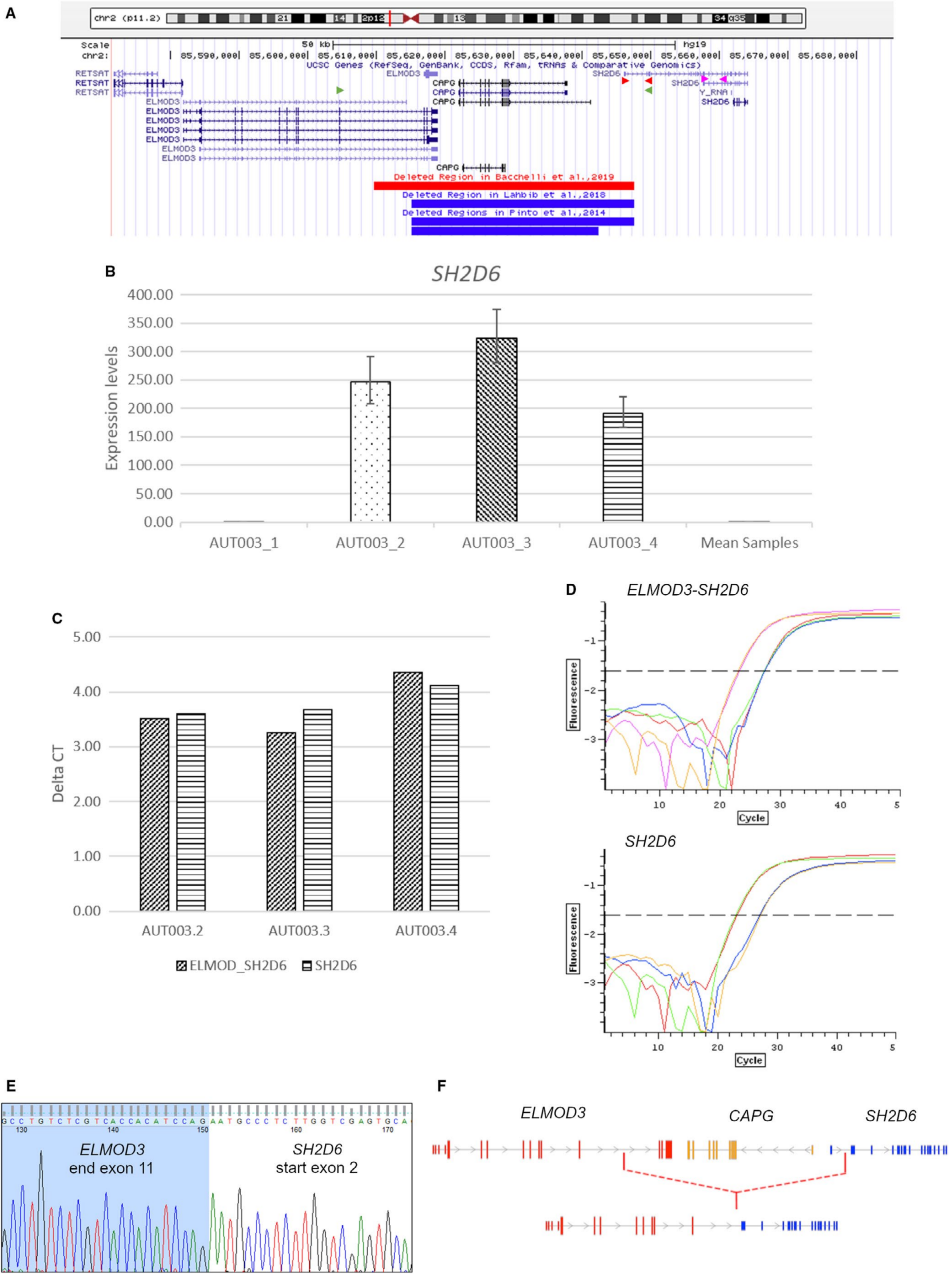
2p11.2 microdeletion and *ELMOD3*‐*SH2D6* gene fusion detection. A, Schematic representation of the microdeletion identified in AUT003 family in the present study and in other three ASD families. Red arrows indicate the primers used in the first gene expression assay (*SH2D6* exon 1 [forward] and *SH2D6* exons 2‐3 [reverse]). Purple arrows indicate the primers used in the second gene expression assay (*SH2D6* exons 11‐12 [forward] and exons 13‐14 [reverse]). Green arrows indicate the primers used for the fusion transcript (*ELMOD3* exon 10 and *SH2D6* exon 2‐3); B, *SH2D6* gene expression assay in AUT003 family members and in controls. Bar plots represent fold change with upper/lower limits relative to controls; C, *ELMOD3*‐*SH2D6* fusion transcript and *SH2D6* gene expression detection in AUT003 family members and in controls. Bar plots represent delta Ct; D, Example of *ELMOD3*‐*SH2D6* transcript fusion and *SH2D6* (primers used in the second assay) qRT‐PCR curves obtained from one sample carrying the deletion. Curves on the left part of the figure refer to the reference gene (*TFRC)* and curves on the right part refer to the transcripts of interest; E, Sequence electropherogram showing the point of fusion between *ELMOD3* exon 11 and *SH2D6* exon 2; F, Schematic representation of the fusion transcript

We conducted a comprehensive genetic analysis of a Sardinian family (AUT003) consisting of two siblings affected by ASD and their unaffected parents.^9^ Genetic studies conducted on Sardinian population have been proven fundamental for the identification of genetic loci linked to several complex diseases.^10^, ^11^, ^12^, ^13^ We identified a rare heterozygous 2p11.2 deletion, including the last coding exons of *ELMOD3*, the entire *CAPG* gene and the first non‐coding exon of *SH2D6*, in both ASD siblings inherited from their unaffected mother (Figure 1A). Interestingly, two similar heterozygous 14 and one homozygous 15 deletions have been reported in three independent ASD families, supporting the clinical relevance of this locus for ASD (Figure 1A).

*ELMOD3* and *SH2D6* genes are transcribed in the same direction, thus the deletion might have generated a gene fusion as hypothesized by Lahbib et al.^15^ Since copy number loss upstream a gene can affect its expression, this work aims to explore the impact of this gene deletion on *SH2D6* gene expression and the possible formation and expression of a fusion transcript between *ELMOD3* and *SH2D6* genes in deletion carriers compared to healthy individuals.

## 2 MATERIALS AND METHODS

Given space constraints in the main text, detailed Material and Methods can be found in the Supplementary Material and Methods file.

## 3 RESULTS

**Figure 2:**
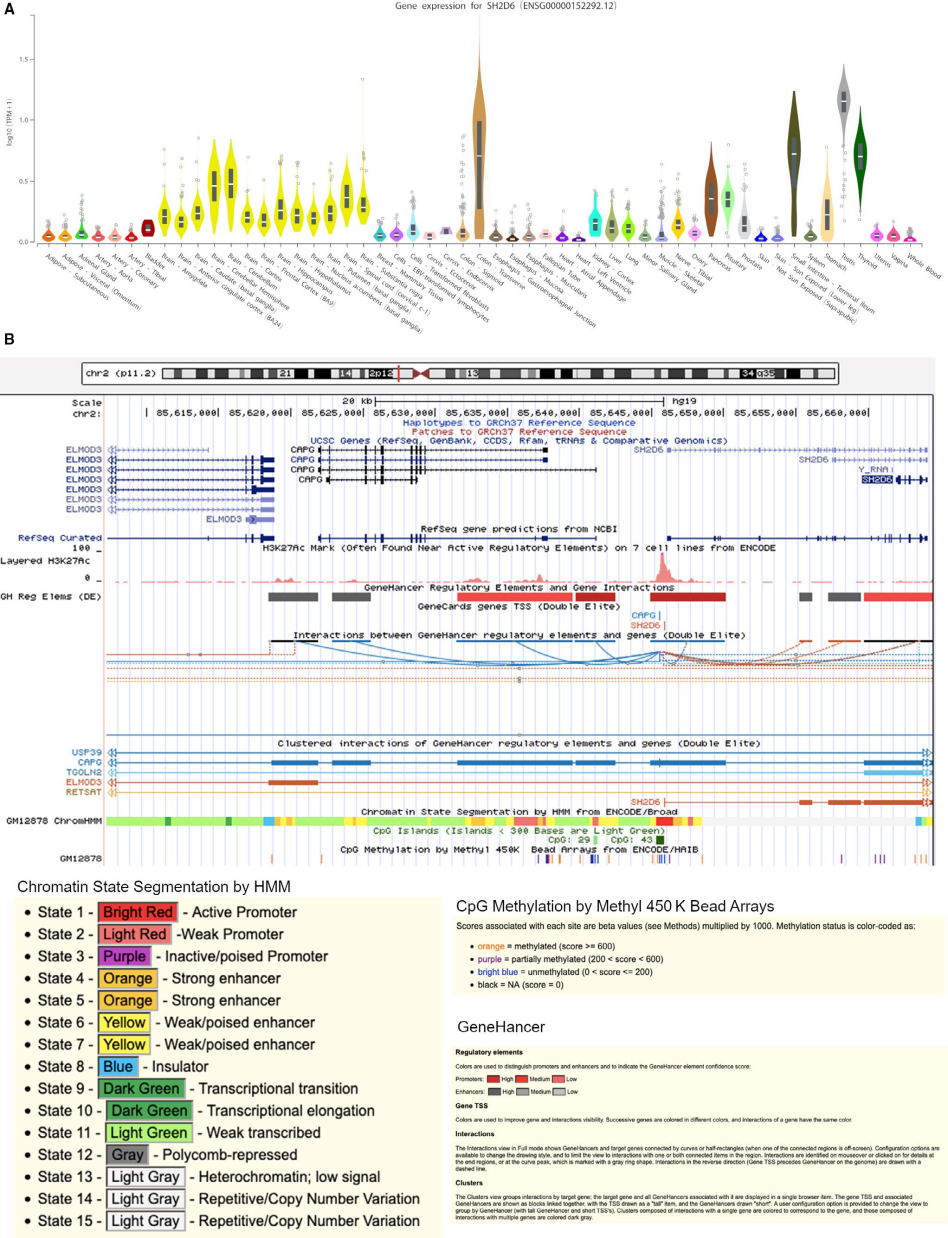
*SH2D6* normal tissues gene expression and regulation analysis. A, *SH2D6* gene expression levels across different tissues (GTEx Data Portal). B, *SH2D6* regulatory region analysis using chromatin state segmentation analysis data from ENCODE/Broad, CpG methylation data from ENCODE/HAIB and GeneHancer data

We evaluated *SH2D6* gene expression in AUT003 family and 17 healthy individuals using two different assays as described in Materials and Methods. The first assay did not reveal any detectable *SH2D6* product in all the tested samples. Since the forward primer maps on the deleted region, the absence of *SH2D6* expression in the individuals with the deletion implies that the wild‐type *SH2D6* locus is not transcribed. In fact, *SH2D6* is expressed at very low levels in whole blood (GTEx data, Figure 2A). The second assay revealed that, as expected, *SH2D6* was undetectable in all subjects without the deletion, including the unaffected father (AUT003.1) (Figure 1B). Interestingly, a significant increase of *SH2D6* mRNA expression levels was detected in the two ASD probands (about 320 folds [*P* < .0001] and 190 folds [*P* < .0001] relative to controls, in AUT003.3 and AUT003.4, respectively), as well as in their unaffected mother (AUT003.2, about 245 folds [*P* < .0001]; Figure 1B).

To understand the reasons why *SH2D6* becomes highly expressed in PBMCs of subjects carrying the deletion, we explored two possible mechanisms: (a) loss of a silencer or an insulator in the deleted region upstream *SH2D6*, or acquisition of an enhancer; (b) gene fusion between *SH2D6* and *ELMOD3*.

The loss of the upstream region of *SH2D6* could have disrupted its regulatory landscape affecting its expression. In fact, it is possible that the deletion of a silencer of an insulator, or approaching an enhancer, results in gene up‐regulation. Since we evaluated *SH2D6* gene expression levels in PBMCs, we investigated *SH2D6* regulatory region in GM12878 cell line finding a large number of regulatory elements upstream *SH2D6* (Figure 2B). Interestingly, most *SH2D6* gene region is heterochromatic and methylated, confirming that this gene is not transcribed in GM12878 cell line. GeneHancer did not reveal elements interacting with *SH2D6* in the deleted region (Figure 2B).

We evaluated the possible presence of a transcript fusion between *ELMOD3* and *SH2D6* by PCR.

A PCR product of the expected size was obtained for ASD probands and their unaffected mother while, as expected, no amplification was detected in controls.

The expression levels of this fusion transcript (evaluated using primers mapping in *ELMOD3* exon 10 and *SH2D6* exon 2‐3) were compared to that of *SH2D6* (*SH2D6* exon 13 and exon 14 primers, PCR product: 99bp) in deletion carriers by qRT‐PCR. Delta Cts (Figure 1C) revealed that transcript expression levels detected using both assays do not differ implying that the increased expression of *SH2D6* in subjects with the deletion is only due to this fusion transcript (Figure 1C and 1).

Sequencing analysis confirmed the presence of this fusion transcript in both ASD siblings and their unaffected mother (Figure 1E and 1). ExPASy Translate tool predicted that the cDNA sequence resulted by the fusion of *ELMOD3* and *SH2D6* transcript would encode for a protein of 258 amino acids (aa), including the first 246 aa of *ELMOD3* and 12 new amino acids encoded by the non‐coding portion of *SH2D6* before a stop codon. Therefore, this chimeric protein would not include the canonical *SH2D6* coding sequence and an interrupted domain of *ELMOD3* (ELMO domain: 170‐324 aa), thus possibly impacting its function. As expected, Western blot for *SH2D6* did not reveal any protein product since the antibody recognizes an epitope which is not present in the predicted chimeric protein sequence (data not shown).

## 4 DISCUSSION

The complex ASD phenotype implied that multiple gene variants participate to ASD phenotypic manifestations. Previous studies have investigated the presence of fusion transcripts in ASD.^6^, ^7^ However, the detected fusion transcripts were present at similar levels in both ASD patients and healthy individual or were expressed at much lower levels compared to wild‐type transcript suggesting that they are subjected to nonsense‐mediated decay. To our knowledge, our study is the first that highlights the possible contribute of a chimeric gene in ASD phenotype. In fact, the so evident and detectable expression of the transcript produced by the identified fusion gene in the cells of deletion carriers suggests that this transcript does not undergo mechanisms of nonsense‐mediated decay. Further investigations will be necessary to reveal the possible translation of a chimeric protein and its possible function, perhaps contributing together with other variants (as already suggested in our previous work, 9) to generate the ASD phenotype.

## CONFLICT OF INTEREST

The authors declare no potential conflicts of interest.

## AUTHOR CONTRIBUTIONS

EL: involved in acquisition and analysis of qRT‐PCR data, statistical analysis, interpretation of data, search strategy, manuscript writing and figures preparation. LM: involved in samples preparation, Sanger sequencing analysis and data interpretation, qRT‐PCR analysis. SB: involved in analysis of qRT‐PCR data, interpretation of data and search strategy. EB: involved in acquisition and analysis of CNV data, qRT‐PCR analysis and supervision, statistical analysis, interpretation of data. AFVB: involved in WB analysis. CC: involved in qRT‐PCR analysis. RF: involved in clinical characterization of patients. EM: involved in search strategy and writing supervision. M.C: involved in clinical characterization. GD: involved in samples collection and clinical characterization supervision. PZ: involved in design and supervision of the study, search strategy supervision, supervision on SNP‐CNV array, qRT‐PCR and sequencing analysis and supervision, interpretation of data, manuscript writing.

## Data Availability

Data generated in this study are available from the corresponding author on reasonable request.
