# Supplementary material for: ELMOD3‐SH2D6 gene fusion as a possible co‐star actor in autism spectrum disorder scenario
Source: J Cell Mol Med. 2019 Dec 4;24(2):2064–9. doi: 10.1111/jcmm.14733 (PMC6991669; doi:10.1111/jcmm.14733)
Supplement: Supplementary file 1 [file JCMM-24-2064-s001.docx]

Supplementary Materials and methods

Participants

AUT003 family consists of two ASD siblings (AUT003.3 and AUT003.4) and their unaffected parents (AUT003.1 and AUT003.2). Clinical features are reported in Bacchelli et al. [9] .

17 additional healthy subjects were recruited from Sardinian population.

qPCR assays and sequencing

Total RNA from all the study participants was extracted from Peripheral Blood Mononuclear Cells (PBMCs) using Qiagen RNAeasy mini kit (Qiagen, Hilden, Germany) and quantified using NanoDrop. cDNA was synthesized using the High Capacity Kit (Applied Biosystems, Carlsbad, CA, USA).

To evaluate *SH2D6* gene expression, two sets of primers have been designed (Figure 1A): the first one mapping on *SH2D6* exon 1 (forward) and *SH2D6* exons 2-3 (reverse) and the second one mapping on *SH2D6* exons 11-12 (forward) and exons 13-14 (reverse). cDNA (50 ng) was amplified by quantitative reverse transcription PCR (qRT-PCR) using SYBR Green. The housekeeping gene *TFRC* was used as reference gene.

To investigate whether the CNV has led to a gene fusion between the 11th exon of *ELMOD3* (ENST00000409013.3) and the 2nd non-coding exon *SH2D6* (ENST00000389938.2), we designed PCR primers closest to CNV breakpoints (mapping in *ELMOD3* exon 10 and *SH2D6* exon 2-3; PCR product: 342 bp) (Figure 1A).

qRT-PCR amplification was conducted in all the subjects carrying the deletion and controls. More than one technical replicates has been performed based on DNA template availability.

The PCR product was purified and subjected to Sanger sequencing.

Public resources

*SH2D6* regulatory region in a B-cell derived cell line, GM12878, was explored using Chromatin State Segmentation analysis data from ENCODE/Broad (https://www.encodeproject.org/). GeneHancer data available in UCSC genome browser (https://genome.ucsc.edu/), which allow to identify enhancers and promoters and uncover their connection with target genes, were investigated.

ExPASy Translate tool (<https://web.expasy.org/translate/>) was used to predict the possible protein sequence of the fusion transcript.
